# Cell-in-cell phenomena across the tree of life

**DOI:** 10.21203/rs.3.rs-3376588/v1

**Published:** 2023-09-29

**Authors:** Stefania E. Kapsetaki, Luis H. Cisneros, Carlo C. Maley

**Affiliations:** 1 Arizona Cancer Evolution Center, Arizona State University, Tempe, AZ, USA; 2 Biodesign Center for Biocomputing, Security and Society, Arizona State University, Tempe, AZ, USA; 3 Tufts University, School of Arts and Sciences, Department of Biology, Medford, MA, USA; 4 School of Life Sciences, Arizona State University, Tempe, AZ, USA; 5 Biodesign Center for Mechanisms of Evolution, Arizona State University, Tempe, AZ, USA; 6 Center for Evolution and Medicine, Arizona State University, Tempe, AZ, USA

## Abstract

Cells in obligately multicellular organisms by definition have aligned fitness interests, minimum conflict, and cannot reproduce independently. However, some cells eat other cells within the same body, sometimes called cell cannibalism. Such cell-in-cell events have not been thoroughly discussed in the framework of major transitions to multicellularity. We performed a systematic review of 508 articles to search for cell-in-cell events across the tree of life, the age of cell-in-cell-related genes, and whether cell-in-cell events are associated with normal multicellular development or cancer. Out of the 38 cell-in-cell-related genes found in the literature, 14 genes were over 2.2 billion years old, i.e., older than the common ancestor of some facultatively multicellular taxa. Therefore, we propose that cell-in-cell events originated before the origins of obligate multicellularity. Cell-in-cell events are found almost everywhere: across some unicellular and many multicellular organisms, mostly in malignant rather than benign tissue, and in non-neoplastic cells. Thus, our results show that cell-in-cell events exist in obligate multicellular organisms, but are not a defining feature of them. The idea of eradicating cell-in-cell events from obligate multicellular organisms as a way of treating cancer, without considering that cell-in-cell events are also part of normal development, should be abandoned.

## Introduction

Major evolutionary transitions in individuality, by definition, refer to a collective alteration after which the units within the “higher-level” individual have aligned fitness interests, minimal conflict amongst themselves, and cannot reproduce independently anymore. Despite the apparent constraints imposed by this definition, we still find eusocial species and obligate multicellular organisms with (1) chimerism, where genetically distinct units of the “higher-level” individual have unaligned fitness interests (unless both genetically distinct units pass into the next generation)^1^; and (2) genetically related units of the group eating each other, and thus displaying significant relational conflict. In the cellular context relevant to multicellularity, this second type of phenomenon is generally called “cell-in-cell events” and typically involves the engulfment of one cell by another. Why and where across the tree of life do we see cell-in-cell phenomena? Are they associated with a ‘selfish’ phenomenon like cancer? And were cell-in-cell phenomena required for, or just incidental to, the transition to obligate multicellularity?

There are over ten definitions of cell-in-cell phenomena and many categorizations mostly based on functional traits^2^ (Table 1; also see glossary in Fais and Overholtzer^3^). Cell-in-cell phenomena have been previously classified based on whether the internalized cell was dead (phagocytosis), or alive (pathogenic phagocytosis, entosis, emperipolesis, etc)^2,4,5^. Cell-in-cell phenomena have also been defined based on the type of interacting cells: identical (entosis, homogenous cannibalism) or different (emperipolesis, heterogenous cannibalism)^6–8^, and in the case of tumors, tumor cell cannibalism^9,10^. Cell-in-cell phenomena have also been classified according to their initial internalization stage: resembling endocytosis (cannibalism, phagoptosis, and enclysis) or invasion of the ‘prey’ cell into the host (entosis and emperipolesis)^11^. Another classification depends on whether the whole (e.g., phagocytosis) or part(s) (e.g., in trogocytosis, nibbling, cannibalism, and partial phagocytosis) of the prey cell are internalized^12^, also highlighted in Elgar’s 1992 definition of cannibalism “the killing and consumption of either all or part of an individual that is of the same species.”^13^ Other times, cell-in-cell phenomena have been classified entirely by the outcome of the cell-in-cell event: death of the host and/or death of the internalized cell, division of the internalized cell, or exit of the internalized cell from the host cell^14–16^. More recent classifications focus on molecular mechanisms and structures, suggesting a dynamic interaction in which both the engulfing cell and the engulfed ones actively participate^11^.

Conspecific cell-in-cell phenomena can be explained by several fitness benefits to the host cell, prey cell, and potential intracellular viruses (Table 3A). For example, a cell eating another cell can be advantageous to the host cell in times of food scarcity and mitosis^17–21^. Also, if the prey cell is a threat to the host cell, for instance considering immune cells that kill cancer cells, then a cancer cell that eats immune cells would have a selective advantage in comparison to other non-cannibalistic cancer cells in the microenvironment^17,22^. However, no study has yet revealed the range of taxa that display cell-in-cell phenomena, whether cell-in-cell phenomena are linked with cancer across taxa, and/or whether cell-in-cell phenomena preceded the evolution of multicellularity (whether they appeared before or after the evolution of obligate multicellularity).

In this review, we focus on the role of cell-in-cell phenomena in the context of minor (facultative) and major (obligate) transitions in multicellularity^23,24^. Specifically, we include only phenomena of cells internalizing whole cells and not just parts of other cells. We first identify cell-in-cell phenomena across the tree of life and propose a classification based on social evolution. Second, we summarize whether conspecific cell-in-cell phenomena are associated with cancer according to the literature. Third, we examine the association and origins of cell-in-cell phenomena in relation to the evolution of multicellularity, by (i) categorizing cell-in-cell phenomena according to the combination of their degree of ‘selfishness’ and their degree of multicellularity (starting from unicellularity, facultative multicellularity, to obligate multicellularity), and (ii) phylogenetically dating the origin of the genes related to cell-in-cell phenomena in order to place the genes in the evolutionary context of their associated biological functions.

## Methods

In order to find records of cell-in-cell phenomena across the tree of life we used the Arizona State University Library One Search tool, which includes the ASU Library catalog and other online search engines, such as Google Scholar, Mendeley, and JSTOR. We searched for articles using the following keywords:

(entos?s OR “homotypic cell cannibalism” OR “cell cannibalism” OR emperitos?s OR enclysis) AND (vertebrat* OR urochord* OR cephalochord* OR echinoderm* OR protostom* OR cnidaria* OR ascomycot* OR basidiomycot* OR amoebozoa* OR embryophyt* OR chlorophyt* OR rhodophyt* OR stramenopila* OR bacter*)

During January 2022 to January 2023, we also searched for articles that mentioned “*phagocytosis*” and cases of cannibalism specifically in the taxa shown in the phylogenetic tree of Aktipis et al^25^. This led to 352 articles. When an article mentioned “*entos?s*”, “*homotypic cell cannibalism*”, “*cell cannibalism*”, “*emperitos?s*”, and referenced another article, we searched for the original publication and included the original publication in Table 1 if the content was relevant to our search. This method has been previously used when conducting systematic reviews^26^. Searching back for such citations added 156 articles to our list. For a more objective assessment of the literature, two of the authors read several of the articles independently. L.H.C. assessed 249 articles, reading from the oldest to the most recent, and S.E.K. assessed 360 articles, reading from the most recent to the oldest. Out of these, a total of 101 articles were assessed by both L.H.C. and S.E.K. We removed patents, web resources, books, newsletter articles, government documents, book chapters, newspaper articles, conference proceedings, reviews, and non-English articles, which led to 337 articles for further assessment. We also excluded 222 articles that turned out to be about irrelevant topics, review articles that directed us to more relevant articles (specifically to articles with original data), articles with no information about cell-in-cell phenomena in specific taxa, or with no information regarding the fate of the engulfed or host cell in terms of one or both remaining alive after the cell-in-cell event (Supplementary Table).

**Figure 1:**
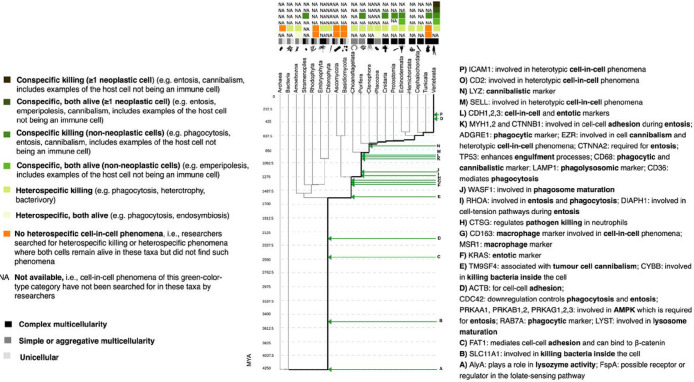
Phylogenetic tree of multicellularity and cell-in-cell phenomena. Adjusted using multicellularity data from^25^,^27^,^146^. We have highlighted in bold the line of how we found the position of each human gene in time from Vertebrates down to the closest common ancestor of all taxa shown in this phylogenetic tree. Made with NCBI Common Tree^147^, iTOL^148^ (version 6.7.4), and PhyloPic (phylopic.org) (version 2.0). Branch lengths show the approximate ages (MYA) of each branch from TimeTree. Detailed information about the genes can be found in Table 2. *No cancer-like phenomena reported.

Our final assessment included 115 articles (Supplementary Table; Supplementary Figure). We collected the following information from these 115 articles: (1) whether the cell-in-cell phenomenon was between heterospecifics or conspecifics; (2) whether the host cell engulfed the whole prey cell; (3) whether both cells remained alive after the cell-in-cell event or at least one cell died; (4) whether both cells were non-neoplastic cells or at least one cell was a neoplastic cell; and (5) the specific taxon of the host cell. We included this information in our across-species comparisons (Table 1; Figure 1).

Our third aim was to examine associations between cell-on-cell phenomena and the evolution of multicellularity across 20 taxa based on previous scales of multicellularity^25,27^. We performed the following ordinal categorical scaling: we categorized taxa in Table 1A according to their multicellularity levels as “unicellular” [0], “simple or aggregative multicellularity” [1], or “complex multicellularity” [2]. If several such multicellularity levels were found in a taxon, we assigned that taxon to the highest reported level of multicellularity. We also categorized cell-in-cell phenomena from Table 1A based on the level of ‘selfishness’ of the interacting cells. The cell-in-cell categories were “no cell-in-cell phenomena reported/found” [0], “heterospecific cell-in-cell phenomena where both cells remain alive” [1], “heterospecific cell-in-cell phenomena where at least one cell dies” [2], “conspecific cell-in-cell phenomena where both cells remain alive” [3], “conspecific cell-in-cell phenomena where at least one cell dies” [4], “conspecific cell-in-cell phenomena where both cells remain alive and at least one of the cells is a neoplastic cell” [5], “conspecific cell-in-cell phenomena where at least one cell dies and at least one of the cells is a neoplastic cell” [6]. If several such cell-in-cell categories were found in a taxon, we assigned that taxon to the highest reported ‘selfishness’ index.

### Gene functional information

Within the 115 articles shown in Table 1, we searched for any mentioned markers of entosis, cannibalism, phagocytosis, emperitosis, and emperipolesis. We collected the names of the cell-in-cell-related genes and information about their cell-in-cell-related function from these articles.

### Gene age data

We found the human homologs of the cell-in-cell-related genes that we had identified (Table 2) and obtained their evolutionary age using a human gene age database published in previous work^28^. These gene ages are determined as the maximum phylogenetic divergence time between humans and the species represented in each gene ontology, as given in the TimeTree database^29,30^. We could not find human homologs of two cell-in-cell-related genes (AlyA and FspA)^31^.

AlyA is a gene found in *Klebsiella pneumoniae*, a gram-negative Enterobacterium. This gene encodes for Alginate lyase, and it is also found in brown (Phaeophyceae) and red algae (Rhodophyta)^32^, which leads to an age of at least 4250 MYA based on the estimated time of divergence^30^.

FspA is a gene found in *Campylobacter jejuni*, also a gram-negative bacterium. It encodes for the Type 3 secretion system protein^33^ and it is found in a great number of eubacteria^34^. This indicates that this gene most likely emerged around 4250 MYA with the evolution of bacteria^30^.

## Results

Cell-in-cell phenomena have been found in 16 taxonomic groups across the tree of life. Cell-in-cell phenomena across the seven different phyla examined in our study can be separated into six different categories from the perspective of social evolution (Fig. 1; Table 1):

1. Heterospecific killing between non-neoplastic cells where at least one of the resulting cells dies, is the most common phenomenon out of the six categories of cell-in-cell phenomena appearing in all 7 examined unicellular, facultative or obligately multicellular phyla (Fig. 1; Table 1).
2. Conspecific killing between non-neoplastic cells where at least one of the resulting cells dies is the second most common phenomenon appearing in three out of the seven examined unicellular, facultative, or obligately multicellular phyla (Fig. 1; Table 1).
3. Heterospecific cell-in-cell phenomena between non-neoplastic cells where both of the cells remain alive have been found in three out of the seven examined unicellular or facultatively multicellular taxa (Fig. 1; Table 1).
4. Conspecific cell-in-cell phenomena between non-neoplastic cells where both of the cells remain alive have been found in one out of the seven examined obligately multicellular phyla (echinoderms) (Fig. 1; Table 1).

In the domains of archaea and bacteria, only heterospecific killing between non-neoplastic cells has been found in bacteria where at least one of the resulting cells dies. Across the four examined divisions of Ascomycota, Basidiomycota, Chlorophyta, and Rhodophyta, only the Chlorophyta have a form of cell-in-cell event which is heterospecific killing between non-neoplastic cells where at least one of the resulting cells dies. Across the two examined clades of stramenopiles and embryophyta, the former have conspecific killing between non-neoplastic cells where at least one of the resulting cells dies, whereas the latter have heterospecific killing between non-neoplastic cells where at least one of the resulting cells dies. Across the three examined subphyla of tunicates, cephalochordata, and vertebrates, the tunicates have conspecific killing between non-neoplastic cells where at least one of the resulting cells dies, the cephalochordata have heterospecific killing between non-neoplastic cells where at least one of the resulting cells dies, and all cell-in-cell categories exist in vertebrates except for heterospecific cell-in-cell events between non-neoplastic cells where both of the resulting cells remain alive (Fig. 1; Table 1).

In many taxa out of the 20 (Fig. 1), many of the above phenomena have not been found or have not been searched for (Table 1A). Within vertebrates, cell-in-cell phenomena have been described in eight species (Table 1B).

Conspecific cell-in-cell phenomena also occur between non-neoplastic cells and appear in multicellular organisms that have no known cancer or cancer-like development. According to the literature (Table 1), there are several examples of cell-in-cell phenomena between non-neoplastic cells. Also, porifera display conspecific cell-in-cell phenomena but have no known cancer-like growth (Table 1). An important caveat here is that the fact that no cancer-like phenomena have been reported in these taxa does not mean that they do not get cancer. They may not have been adequately studied yet to know if they can get cancer^25^.

Cell-in-cell phenomena do not appear to be associated with the levels of multicellularity, and we were able to identify 38 cell-in-cell-related genes, some of which appeared before the origins of obligate multicellularity (Fig. 1). Fourteen cell-in-cell-related genes originated over 2.2 billion years ago. These genes are AlyA, FspA, SLC11A1, FAT1, LYST, ACTB, CDC42, PRKAA1, PRKAB1,2, PRKAG1,2,3, and RAB7A (Fig. 1; A–D). They are related to the AMPK pathway, folate-sensing pathway, cell-cell adhesion, entosis, phagocytosis, intracellular bacterial killing, cell cannibalism, lysozyme activity, and lysosomal maturation (Table 2). 24 cell-in-cell-related human genes originated after the common ancestor of amoeba and humans. These genes (Fig. 1; E–P) are related to cell-in-cell phenomena such as cell-cell adhesion, entosis, macrophage function, phagocytosis, killing pathogens, phagosome maturation, and cell cannibalism. TM9SF4 is a gene related to tumor cell cannibalism. KRAS is a gene related to entosis. CD163, MSR1, and CTSG are genes related to killing pathogens. DIAPH1 is a gene related to entosis, and RHOA is a gene related to entosis and phagocytosis. Homologs of MYH1,2 and CTNNB1 are involved in cell adhesion during entosis, CDH1,2,3 are involved in entosis, WASF1, ADGRE1, and LAMP1 are related to phagocytosis, EZR is related to cell cannibalism and heterotypic cell-in-cell phenomena, CTNNA2 is required for entosis, TP53 is required for cell engulfment, CD36 is involved in phagocytosis, CD68 is involved in phagocytosis and cell cannibalism, and SELL is related to heterotypic cell-in-cell phenomena. LYZ is a gene related to cell cannibalism. Two cell-in-cell-related human genes, CD2 and ICAM1, originated in the taxon of vertebrates around 313–400 million years ago (Fig. 1; O–P). These genes are involved in entosis, heterotypic cell-in-cell phenomena, and phagosome maturation. No cell-in-cell phenomena have been reported in archaea, red algae, ascomycota, and basidiomycota, even though homologs of 14 cell-in-cell-related genes could be present in the red algae (Fig. 1; A–D) and homologs of 20 cell-in-cell-related genes could be present in the ascomycota and basidiomycota (Fig. 1; A–H).

## Discussion

This study led to four main findings. First, cell-in-cell phenomena are present in at least 16 different taxonomic groups on the tree of life. These cell-in-cell phenomena can be classified into six useful categories from the perspective of social evolution that depend on the degree of relatedness between host and prey (conspecifics or heterospecifics), the outcome of the event (whether both or only one cell remains alive after the cell-in-cell phenomenon), and the type of cells (neoplastic or non-neoplastic cells). Second, conspecific cell-in-cell phenomena occur in neoplastic cells as well as non-neoplastic cells. Third, we did not find any significant association between cell-in-cell phenomena and the levels of multicellularity. Fourth, many cell-in-cell genes evolved before the origins of obligate multicellularity.

### Cannibal neoplastic and non-neoplastic cells

Cell-in-cell phenomena happen between neoplastic and non-neoplastic cells. Depending on the details of the cell interaction, the outcome of cannibalism can be either the growth or shrinkage of the neoplasm. Thus, cannibalism cannot be considered a characteristic of only uncontrollably dividing ‘selfish’ neoplastic cells.

Due to their relatively higher nutritional demands, neoplastic cells are often cannibals^159^. Cell-in-cell phenomena can lead to aneuploidy and/or tetraploidy, and more aggressive and invasive tumors^59,160^. Therefore, it is no surprise that cannibalism is more often found in malignant than benign biopsies and urine cytology samples^161,162^, in metastatic tumors than in primary melanoma^17^, in aggressive than in non-aggressive giant cell granuloma^163^, in grade 3 rather than in grade 1 urothelial carcinoma^164,165^, in higher rather than in lower grade breast tumors^166^, and in higher rather than in lower grade superficial papillary cancer^167^. Still, finding cell-in-cell phenomena together with malignancy does not necessarily mean that cell-in-cell phenomena induce malignancy, neither it is sufficient nor necessary for malignancy. In fact, cell-in-cell phenomena may be a mechanism to suppress malignancy. For example, entosis was shown to suppress tumor growth in breast cancer cell lines^73^, and breast cancer cells that have cannibalized mesenchymal stem/stromal cells were shown to enter a dormant state^93^.

Cell-in-cell phenomena occur outside of the context of cancer. *Bdellovibrio* bacteria were observed to enter and kill other Gram-negative bacteria^143^ without any evidence of preference for killing ‘cheating’ bacteria. Poriferan cells perform conspecific cell-in-cell phenomena without any evidence of cancer-like growth (Fig. 1). Additionally, cell-in-cell phenomena are characteristic of non-cancer cell types during development. Sperm and egg can fuse, forming a zygote. Around 5–6 days after the first cell division of a human embryo, some of the mother’s (uterine epithelial) cells enter the fetus (trophectoderm) cells^56^. We can assume that this process reduces the chances of fetal cells being rejected by the mother. In *Caenorhabditis elegans*, endodermal cells eat parts of primordial germ cells^168^. In *Xenopus laevis*, endoderm cells can engulf their own as well as their neighbor’s membrane^169,170^. In *Drosophila*, mice^171^, zebrafish^172,173^, and humans, later in development, multinucleated cells can form when myoblast cells fuse with myoblast cells^67^, osteoclast cells fuse with other osteoclast cells, and hepatocyte cell fuse with other hepatocyte cells^68^. Phagocytosis is also a common mechanism for the immune system to discard pathogenic foreign cells, as well as cells from the same organism that have been programmed for disposal (Table 1).

### Origins of cell-in-cell-related genes

We searched for associations between 38 cell-in-cell-related genes and the origins of obligate multicellularity, as well as correlations between the levels of ‘selfishness’ of cell-in-cell phenomena and the level of multicellularity, but did not find any significant associations. This shows that, mechanistically, cell-in-cell phenomena likely predate the evolution of obligate multicellularity. A gene worth mentioning reminds us of Charles Darwin’s saying about how development can teach us about evolution: the transmembrane 9 superfamily protein member 4 gene TM9SF4^174^ in the obligately multicellular humans encodes an ion channel, possibly regulating the pH of intracellular vesicles of malignant cells^83^. This gene is relatively highly expressed in the placenta relative to other tissues^175^. If this is true across mammals, this gene might play a role in two phenomena. First, among mammals, Carnivora only have the endotheliochorial placental type (where some of the mother’s uterine epithelial cells have entered trophectoderm cells of the blastocyst via entosis^56^)^176^. Second, among mammals, Carnivora have the highest cancer prevalence/risk^177–179^. Since the origin of this gene, ~1.5 billion years ago, another gene phg1A, a homolog of TM9SF4, has been found in facultatively multicellular extant amoeba *Dictyostelium discoideum* playing a role in phagocytosis^9,83,180^. Thus, TM9SF4 and its homolog have a history in cell-in-cell phenomena before and after the evolution of obligate multicellularity.

### Limitations

Despite observations in the literature that cell-in-cell phenomena exist in both benign and malignant neoplasms as well as during normal development, cell-in-cell phenomena have not been searched for in many species across the tree of life.

In this article we mention the cell-in-cell-related functions of genes dating back to the last common ancestor between humans and the corresponding current non-human taxon that carries that gene. Even though the function of genes may have changed over billions of years, functional annotations (Table 2) suggest that they are associated with these phenomena in humans as well.

Understanding what drives the forms of cell-in-cell phenomena that suppress tumor growth versus those that enhance tumor growth could help the management of cancer. For example, following a calorie-restricted diet^181^ or using caloric restriction mimetics in clinical trials^182^ may not necessarily be a successful strategy to treat cancer considering that starvation drives cell-in-cell phenomena (Table 3), and cell-in-cell phenomena can lead to both tumor suppression and tumor progression^59,73,183^. Therefore, understanding the difference in conditions that determine one outcome versus the other would be of principal importance, and potentially of benefit for treatment based on a precision medicine approach. Interestingly, exosomes can be ingested by cancer cells^184^. The process of internalization of exosomes into cells occurs in a similar way to endocytosis^185^ or phagocytosis^186^. However, serving cannibalistic cancer cells a ‘ticking bomb’ of exosomes full of drugs may not be a successful anticancer strategy either, as these cells can also ‘spit out’^76^ exosomes. Mapping cell-in-cell-related genes onto phylogenies and homology families using appropriate criteria of evolutionary relatedness that take into account cell-in-cell phenomena, disentangling the corresponding genetic networks, ascertaining the role of possibly antagonistic pleiotropic cell-in-cell-related genes, their expression patterns before and after reproductive age, and exploring the micro-/macro-environmental triggers that might change the risk of cell-in-cell events suppressing or promoting cancer, will be major cuts through the ‘Gordian knot’ in understanding the evolutionary history of multicellularity, cancer, and cell-in-cell phenomena across species.

### Open question: connection between microscopic and macroscopic cannibalistic phenomena

There may also be connections between microscopic and macroscopic cannibalistic phenomena. Factors that drive cannibalism in the microscopic world (micrometer scale) may also drive cannibalism in the macroscopic world (centimeter or meter scale). Examples of such analogies can be seen in Table 3. Under starvation, both cells and obligate multicellular organisms (e.g., honeybees, mantises, and mice) perform cannibalism. Mammals with large litter sizes also cannibalize their young in periods of food scarcity^187^. Under attack by the enemy (immune system or specific tribe), cancer cells and humans (respectively) cannibalize their enemy. Upon landing in a new environment, entotic uterine cells and cannibalistic beetles are more likely to survive (Table 3). However, no one has yet quantitatively estimated all the different factors (natural selection, random genetic drift, mutation, migration) that may drive cannibalistic processes both at the microscopic and macroscopic scales.

### Conclusion

Overall, this study is the first to systematically analyze cell-in-cell phenomena across the tree of life. Here we provide a classification of cell-in-cell phenomena using a social evolution perspective, in the context of evolutionary transitions. This work highlights how cell-in-cell phenomena can be organized by the degree of relatedness, survival or death outcome of the interacting cells, and ‘selfish’ overall/cancerous behavior, in terms of the fitness outcome (survival), of the interacting cells. This study also highlights that cell-in-cell phenomena are not only found among cancer cells but also among benign neoplastic and normal cells. Cell-in-cell phenomena are not a definitive feature of obligate transitions to multicellularity, given that cell-in-cell genes have been found even before the origins of facultative multicellularity. Thus, the prerequisite for minimal conflict in the definition of major transitions in multicellularity refers to ‘conflict’, as in the presence of cell-in-cell phenomena within a broad range of multicellular organisms, and ‘minimal’, as in not all cells of multicellular organisms eat each other.

## Figures and Tables

**Table 1A.** Cell-in-cell phenomena across the tree of life. We obtained data on the levels of multicellularity and cancer from Aktipis et al.^25^ and Fisher et al.^27^. In Archaea, even though an endosymbiosis event involving an Archaeon is widely acknowledged as the event which led to the origin of eukaryotes, there is no evidence of phagocytosis currently happening in Archaea^35^. Similarly, in other taxa, we only present evidence of cell-in-cell phenomena from recently observed events. NA: not available.

Taxon (ranking)	Level of		Cell engulfs heterospecific cell & kills the engulfed or host cell	Cell engulfs heterospecific cell & both cells remain alive	Cell engulfs conspecific cell & kills the engulfed or host cell & both are non-neoplastic cells (includes examples of the host cell not being an immune cell)	Cell engulfs conspecific cell & both cells remain alive & both are non-neoplastic cells (includes examples of the host cell not being an immune cell)	Cell engulfs conspecific cell & kills the engulfed or host cell & ≥1 is a neoplastic cell (includes examples of the host cell not being an immune cell)	Cell engulfs conspecific cell & both cells remain alive & ≥1 is a neoplastic cell (includes examples of the host cell not being an immune cell)

	Multicellularity	Cancer						

Vertebrata	Complex	Cancer	✔	NA	✔	✔	✔^72^	✔^72^
(subphylum)	multicellularity	reported	(phagocytosis)^36–44^		(phagocytosis)^43,45–50^ & (cannibalism)^51,52^ & (entosis)^53–61^ & (efferocytosis)^62^ & (emperipolesis)^63^ & (phagoptosis)^64,65^ & (fusion)^66–68^	(emperipolesis)^38,69–71^ & (entosis)^55^	(cannibalism)^2,9,17,18,73–83^ & (entosis)^59,73–75,84–88^ & (engulfing)^89–93^ & (cell-in-cell)^94–96^ & (internalization)^97^ & (phagocytosis)^22,98–106^ & (fusion)^107,108^	(entosis)^73^ & (emperipolesis)^101^ & (cannibalism)^2^ & (internalization)^97^ & (engulfment)^109^ & (cell-in-cell)^96^
Urochordata/Tuni cata (subphylum)	Complex multicellularity	Cancer reported	☓ (no reported phagocytosis)^110^	☓ (no reported phagocytosis)^110^	✓ (trephocytes within the egg)^111,112^	NA	NA	NA
Cephalochordata (subphylum)	Complex multicellularity	Cancer reported	✔ (intracellular digestion)^110^	NA	NA	NA	NA	NA
Echinodermata (phylum)	Complex multicellularity	Cancer reported	(intracellular digestion)^110^	NA	✓ (cannibalism)^113^	✓ (cannibalism)^113^	NA	NA
Hemichordata (phylum)	Complex multicellularity	No cancer-like phenomena reported	(intracellular digestion)^110^	NA	NA	NA	NA	NA
Protostomia (unranked)	Complex multicellularity	Cancer reported	✔ can be dying cells (phagocytosis)^114—117^	NA	✔ (phagocytosis)^118–120^ & (fusion of gonad-to-cloaca cells via entosis and then death of the entosed cell)^121^ & (eating nutrient from a dead conspecific)^118^ & (trephocytes within the egg)^111,112^	NA	NA	NA
Cnidaria (phylum)	Complex multicellularity	Cancer reported	✔ (phagocytosis)^122,123^	NA	✔ (nurse cells transfer cytoplasm to oocyte via ring canals, they undergo apoptosis and then phagocytosis)^124^	NA	NA	NA
Placozoa (phylum)	Simple/aggregative multicellularity	No cancer-like phenom ena reported	✔ (“phagocytos is may be present”)^110^	✔ (“phagocytos is may be present”)^110^	NA	NA	NA	NA
Porifera (phylum)	Simple/aggregative multicellularity	No cancer-like phenomena reported	✔ (phagocytosis)^123,125–130^	NA	✔ (oocytes phagocytose nurse cells)^131,132^	NA	NA	NA
Ctenophora (phylum)	Complex multicellularity	No cancer-like phenom ena reported	(intracellular digestion)^110^ & (phagocytosis)^123^	NA	NA	NA	NA	NA
Choanoflagellata (class)	Simple/aggregative multicellularity	No cancer-like phenom ena reported	✔ (intracellular digestion)^110^ & (phagocytosis)^133^	NA	NA	NA	NA	NA
Ascomycota (division)	Unicellularity, simple or aggregative multicellularity, complex multicellularity	Cancer reported	☓ (“no phagocytosis has been reported”)^134^	☓ (“no phagocytosis has been reported”)^134^	NA	NA	NA	NA
Basidiomycota (division)	Unicellularity, simple or aggregative multicellularity, complex multicellularity	Cancer-like phenomena reported	☓ (“no phagocytosis has been reported”)^134^	☓ (“no phagocytosis has been reported”)^134^	NA	NA	NA	NA
Amoebozoa (phylum)	Unicellularity, simple or aggregative multicellularity	Cancer-like phenom ena reported	✓ (phagocytosis)^31,135–138^	✓ (endosymbiosis)^135^	NA	NA	NA	NA
Embryophyta (clade)	Complex multicellularity	Cancer reported	☓ whole (endocytosis)^139^	NA	NA	NA	NA	NA
Chlorophyta (division)	Unicellularity, simple or aggregative multicellularity, complex multicellularity	Cancer-like phenom ena reported	☓ whole (bacterivory)^140^	NA	NA	NA	NA	NA
Rhodophyta (division)	Unicellularity, simple or aggregative multicellularity, complex multicellularity	Cancer reported	☓ (no known phagocytosis)^141^	☓ (no known phagocytosis)^141^	NA	NA	NA	NA
Stramenopiles (clade)	Unicellularity, simple or aggregative multicellularity, complex multicellularity	Cancer-like phenomena reported	NA	NA	(cannibalism)^142^	NA	NA	NA
Archaea (domain)	Simple/aggregative multicellularity	NA	☓ (no known phagocytosis)^35^	NA	NA	NA	NA	NA
Bacteria (domain)	Unicellularity, simple or aggregative multicellularity	Cancer-like phenom ena reported	✔ (cell-in-cell)^143^	NA	NA	NA	NA	NA

**Table 1B.** Cell-in-cell phenomena in vertebrate species. All the species below perform heterospecific killing (phagocytosis), but none perform heterospecific cell-in-cell phenomena where both cells remain alive.

Species	Cell engulfs conspecific cell & killing of the engulfed or host cell & both are non-neoplastic cells	Cell engulfs conspecific cell & both cells remain alive & both are non-neoplastic cells	Cell engulfs conspecific cell & killing of the engulfed or host cell & ≥1 is a neoplastic cell	Cell engulfs conspecific cell & both cells remain alive & ≥1 is a neoplastic cell

*Danio rerio*	✔ (entosis)^54^	(emperipolesis)^38^	NA	NA
*Sparus aurata*	✔ (phagocytosis)^40^	NA	NA	NA
*Pelodiscus sinensis*	✔ (entosis)^53^	NA	NA	NA
*Rattus norvegicus*	✔ (phagocytosis)^45^	NA	✔^72^	✔^72^
*Mus musculus*	✔ (phagocytosis)^43,45–47,50^ & (entosis)^55–57^ & (emperipolesis)^63^ & (phagoptosis)^65^	✔ (entosis)^55^	✔ (engulfment)^89^	NA
*Felis catus*	✔ (phagocytosis)^144^	NA	✔ (cannibalism)^75^	NA
*Canis lupus familiaris*	✔ (phagocytosis)^44,145^	NA	✔ (cannibalism)^75^	NA
*Homo sapiens*	✔ (cannibalism)^51,52^ & (phagocytosis)^48–50^ & (efferocytosis)^62^ & (entosis)^58–61^ & (engulfment)^48^ & (fusion)^66^	✔ (emperipolesis)^69–71^	✔ (engulfment)^89–93^ (entosis)^74,84–88^ & (internalization)^97^ & (cannibalism)^9,17,18,73,74,76–83^ & (cell-in-cell)^94–96^ & (phagocytosis)^22,98–106^ & (fusion)^107,108^	✔ (internalization)^97^ & (emperipolesis)^101^ & (engulfment)^109^ & (cell-in-cell)^96^

**Table 2.** Genes involved in cell-in-cell phenomena. The genes are listed in the table from youngest to oldest. We found these cell-in-cell-related genes from our systematic search of the literature. MYA: million years ago.

Markers/regulators of cell-in-cell phenomena (mentioned in reference column)	Organism(s) (to which the marker/regulator in the 1st column belongs)	Cell-in-cell related function(s) as mentioned in the reference(s)	Reference	Human homologue	Cell-in-cell related function(s) of the human homologue	Age of the human homologue gene (MYA)

ICAM-1	humans	involved in forming heterotypic cell-in-cell structures	^96^	ICAM1	–	361.8
LFA-2	humans	involved in forming heterotypic cell-in-cell structures	^96^	CD2	–	400.1
lysozyme	humans	cannibalistic marker	^149^	LYZ	–	762.8
CD62L	humans	involved in forming heterotypic cell-in-cell structures	^96^	SELL	–	891.7
E-cadherin	humans, cats, dogs	marker of cell-in-cell structures, including cannibalizing and entotic cells; required for homotypic cell-in-cell structure formation; forms concentrated foci at contact points between cells and is required for entosis; required for the formation of adhesive junctions between cells during entosis	^3,55,59,61,73,75,84,87,89,96,97,109,150^	CDH1	–	937.4
N-cadherin	humans	aids in the formation of cell contacts required for homotypic cell-in-cell formation; a cell-cell adhesion molecule that facilitates cell-in-cell invasion	^85^	CDH2	–	937.4
P-cadherin	humans	required for cell-cell adhesion and cell uptake during entosis	^3,73^	CDH3	–	937.4
F4/80	humans	macrophage and phagocyte marker	^56,79^	ADGRE1	–	940
Crq/Dsb	*Drosophila*	related to phagosome maturation	^49^	CD36	mediates phagocytosis (e.g., phagocytosis of *Plasmodium falciparum*)^151^	940
CD68	humans	phagocytic, macrophage, and cannibalistic marker expressed by cannibal and internalized cells	^79,80,82,91,99,149,152^	CD68	–	940
a-catenin	humans	core adhesive component indispensable for entosis	^74^	CTNNA2	–	940
β-catenin	humans	required for cell-cell adhesion; required for cell internalization during homotypic cell-in-cell phenomena; required for forming adhesive junctions and is gradually degraded during entosis	^61,87,96,109^	CTNNB1	–	940
ezrin	humans	a cell cortical protein involved in forming heterotypic cell-in-cell structures, including cannibalism	^3,96^	EZR	–	940
LAMP, LAMP1	*Drosophila*, turtles, humans	phagolysosome marker expressed by epidermal cells	^49,58^	LAMP1	–	940
actomyosin	humans	required for cell-cell adhesion prior to ingesting neighboring live cells	^153^	MYH1	–	940
myosin	zebrafish	drives the initial steps of entosis	^54,153,154^	MYH2	–	940
p53	humans	mutant p53 increases cellular engulfment leading to cell-in-cell phenomena	^109^	TP53	–	940
WASH	*Dictyostelium*	involved in vesicular trafficking and phagosome maturation	^31^	WASF1	cell-in-cell related (e.g., “receptor- mediated endocytosis”)	1184.5
DIA1	humans	regulates cell tension	3	DIAPH1	–	1215.8
Rho, RhoA	metazoa	regulates actin polymerization required for cell-cell adhesion, contractility of the internalized cell, phagocytosis, and entosis	^54,90,109,119,134,153,155,156^	RHOA	–	1215.8
cathepsin G	mice	regulates the ability of neutrophils to kill *Staphylococcus aureus* pathogens	^31^	CTSG	–	1362
CD163	humans	marker of macrophages involved in cell-in-cell phenomena	^91^	CD163	–	1369
CD204	humans	“M2-polarization macrophage marker”	^107^	MSR1	–	1369
K-Ras	humans	molecular marker of entosis	^109^	KRAS	–	1381.2
Nox2	mammals	involved in intracellular killing of bacteria	^31^	CYBB	–	1530.3
TM9SG4, Phgla	humans, *Dictyostelium*	associated with tumor cell cannibalism; controls “intracellular transport and stability of membrane proteins” and intracellular killing	^31,157^	TM9SF4	–	1530.3
actin	zebrafish	required for cell-cell adhesion prior to the ingestion of neighboring live cells	^38,153^	ACTB	–	2269.5
Cdc42	bacteria, humans	regulates actin polymerization and phagocytosis; required for entosis	^54,74,134,155^	CDC42	–	2269.5
LvsB	*Dictyostelium*	involved in lysosome maturation	^31^	LYST	regulates phagosome maturation and lysosome organization^158^	2269.5
AMPK	humans	regulates and is required for entosis; glucose “suppresses entosis induction by inhibiting activity of the energy-sensing AMP-activated protein kinase (AMPK)”; cells “with the lowest energy levels, and concomitantly the highest levels of AMPK activity, are sacrificed to feed those with lowered AMPK activity”	^3^	PRKAA1 PRKAB1 PRKAB2 PRKAG1 PRKAG2 PRKAG3	–	2269.5
Rab7a	*Drosophila,* humans	phagocytic and late phagosome/phagolysosome marker	^49,99^	RAB7A	–	2269.5
FAT1	humans	mediates cell-cell adhesion	^51^	FAT1	–	2535.8
NRAMP1	*Dictyostelium*, mammals	a metal ion transporter on the phagosomal membrane of mammalian macrophages involved in intracellular bacterial killing	^31^	SLC11A1	–	3556.3
AlyA	*Dictyostelium*	responsible for almost 50% of the total cellular lysozyme activity	^31^	*		4250
FspA	*Dictyostelium*	may act as a receptor or regulator in the folate-sensing pathway	^31^	*		4250

* AlyA and FspA do not have a human homologue (see Methods). In the 6th column we only mention the function of the non-vertebrate genes that have a human homologue.

**Table 3:** Examples of conspecific cell-in-cell behaviors (A), cannibalistic multicellular animal behaviors (B), and the direct fitness benefit to the host, prey, or virus. We have placed examples of A and B that are conceptually similar in the same row but in different columns (separated by a vertical black line).

Event	Direct fitness benefit to			Reference	Event	Direct fitness benefit to			Reference
	predator	prey	virus/prion within prey			predator	prey	virus/prion within prey	

A					B				

examples of cell cannibalism in Vertebrata in Table 1					cannibalism has happened in 16 families of spiders, fall armyworms (*Spodoptera frugiperda*), the American alligator (*Alligator mississippiensis*), 13 avian families, 75 species of mammals [including hedgehogs, voles, mice, woodrats, rabbits, shrews, moles, and 14 species of carnivores such as polar bears, pumas (*Puma concolor*), lynx (*Lynx lynx*), leopards (*Panthera pardus*), and sea lions (*Phocarctos hookeri*)]	+			^187,188^

under hypoxia, acidity, mitosis^74^, and starvation (termed “the great equalizer”^187^, i.e., regardless of relatedness, both kin and non-kin are eaten, expressed as “Ι’m starving, I’ll eat anything!”, and “No one can… love his neighbors on an empty stomach.” - Woodrow Wilson)/hibernation/absence of vasculature in a multicellular body, a cell that eats a related cell has a resource and survival benefit in comparison to a non-cannibalistic cell	+			^17–20,53^	during food scarcity, eusocial species, such as honeybees (*Apis mellifera*), cannibalize their newborn	+			^189^
					during food scarcity, voles, mice, woodrats, rabbits, shrews, moles, and hedgehogs cannibalize their young	+			^187^
					cannibalism between mantises that have been starved	+			^187^
					sibling cannibalism between acorn woodpeckers due to asynchronous hatching	+			^187^
					sibling cannibalism between snowy egret (*Leucophoyx thula*) hatchlings due to unequal provision of hormones to the eggs	+			^187^

the resulting possible change in the shape of the host cell after the cell-incell event^168^ may also help it adapt in the new microenvironment(s) during metastasis or development^190^, e.g., uterine luminal epithelial cells enter blastocyst trophoblast cells during the first few days of pregnancy	+			^56^	cannibalism can be advantageous when flour beetles (*Tribolium castaneum*) colonize new environments	+			^191^
the engulfed cell can also become the host cell’s ‘passport’, or ‘passepartout’, entitled “I am what I eat”. In other words, “having a local inside me helps me pass the border”. For instance a cancer cell fusing with a macrophage and via horizontal gene transfer gaining genetic ‘knowledge’ about a different environment, helping the cancer cell avoid the immune system, become more radioresistant, express the macrophage-specific marker CD163, and become more adapted and ‘blended’/‘accepted’ in the new environment(s) and thus be more metastatic	+			^163,192–196^					

a slime mold cell with the ability to habituate towards a repellent can transfer this trait to the host cell that ate it	+			^197^	humans drank other people’s blood in the 18th century as medicine	+			^187^
similar to a Trojan horse, the engulfed cell (e.g., lymphocyte) can become more toxic inside the host cell (e.g., neoplastic cell) and kill the host cell while the engulfed cell escapes	+			^8,198,199^					

multiple myoblast cells fuse together leading to the formation of multinucleated myoblast cells later in development	+			^67^	adelphophagy or sibling cannibalism between raptors and sand tiger shark embryos (*Carcharias taurus*)	+			^187^

					inability to recognise conspecifics early in development (in cases where kin recognition mechanisms have not yet developed), starvation and lack of other food sources, and overcrowding can lead to cannibalism in clams, insects, and scorpions	+			^187^

a non-digested engulfed cell inside a dormant neoplastic cell is protected from possible attack by other external biological/chemical agents		+		^93^	mouthbrooding in cichlids results in some newborn being eaten	+	+		^187^

a cancer cell that eats immune cells, which are about to eat the cancer cell, has a survival benefit	+			^17,22^	the Aztecs, Caribs, Lendi, Batak, Dyak, Sawney Bean’s clan, Iroquois, Nuuchahnulth, Ancestral Puebloans, Tupinamba, Wari’, Marquesans, Mianmin, Asmat, Fijians, and Maori have practiced exocannibalism, i.e., eating humans that are their enemies	+			^200^

a prey cell enters a host cell and commits apoptosis	+			^11^					

the engulfed genetically related cell may benefit from the predation event if its exact genome is passed to its host’s (relative’s) nucleus and on to the next generation at every host cell division		+		^201–203^	monkeys, rodents, lagomorphs, carnivores, primates, and most artiodactyls eat their placenta after giving birth	+			^187^

Epstein-Barr virus can infect another cell when its host cell is eaten by another cell			+	^204^	cannibalism in humans has happened during famine in ancient Egypt, ancient Greece, ancient Rome, Persia, India, China, Japan, between 793 CE and 1317 CE in Europe, and in the 1950s in Papua New Guinea (which led to the spread of abnormally folded proteins, called prions, causing the fatal disease kuru)	+		+	^187^
